# Lepidiumuridine A: A New Natural Uridine Derivative as a Phytoestrogen Isolated from the Seeds of* Lepidium apetalum* Willd.

**DOI:** 10.1155/2018/2813465

**Published:** 2018-09-04

**Authors:** Meng Li, Mengnan Zeng, Zhi-guang Zhang, Beibei Zhang, Jingke Zhang, Xiaoke Zheng, Weisheng Feng

**Affiliations:** ^1^Henan University of Chinese Medicine, Zhengzhou 450046, China; ^2^Collaborative Innovation Center for Respiratory Disease Diagnosis and Treatment & Chinese Medicine Development of Henan Province, Zhengzhou 450046, China

## Abstract

There has been great interest in phytoestrogens, which are polyhydric compounds that are derived from plants and have a structure similar to that of the mammalian steroid hormone 17*β*-estradiol. The present study examined the estrogenic effects of a new natural uridine derivative, lepidiumuridine A (LA), that was isolated from the seeds of* Lepidium apetalum*. The structure was clarified and determined via analysis of extensive spectroscopic data interpretation. The activity of LA was investigated by measuring the levels of estradiol (E2), luteinizing hormone (LH), follicle stimulating hormone (FSH), and the uterus growth in mice. The proliferation experiment of MCF-7 breast cancer cells was also conducted. Western blot, in-cell western, and antagonist assays with methyl piperidino-pyrazole (MPP) were used for exploring the mechanism of the effects of LA. The results showed that LA elevated the uterine coefficient, the levels of E2, and FSH significantly. In addition, LA significantly elevated ER*α* expression in the uterus and MCF-7 cells. MPP inhibited the proliferation of LA-stimulated MCF-7 cell and ER*α* expression in MCF-7 cells. Taken together, LA had an estrogen-like effect, which was mainly mediated by the estrogen receptor ER*α*.

## 1. Introduction

The seeds of* Lepidium apetalum *Willd. have been thought as traditional Chinese herbal medicine. The use of these seeds was first chronicled in “Shennong's Herba” and described as having cold nature, an acrid and bitter taste. This herb has the ability to relieve asthma and clear away heat from the lungs, detumescence, and promoting diuresis. Furthermore, the dry mature seeds of* L. apetalum* and herb-Sophia (*Descurainia sophia* L.) Webb ex Prantl., belonging to the Cruciferae (Brassicaceae) family, were commonly called “Tinglizi”; the former was called “BeiTinglizi”, and the latter was named “Nan Tinglizi” in the Chinese Pharmacopoeia 2015 [1]. Although they have been both used as “Tinglizi”, distinctions have been made between their plant origin, appearance, and pharmacological activity [2, 3]. Compared with the seeds of* L. apetalum*, the previous investigations of herb-Sophia semen were more comprehensive [4–9]. Modern pharmacology has demonstrated that the seeds of* L. apetalum *have a cardiotonic [10] effect, and the chemical constituents are mainly flavonoids [11–15].

Phytoestrogens are polyhydric compounds derived from plants and have a similar structure to mammalian steroid hormone 17*β*-estradiol. In our preliminary study, we found that* L. apetalum* exerted estrogen-like effects. In a continuing search for phytoestrogens, various chromatographic techniques were used to isolate a new natural derivative lepidiumuridine A (**1**) from the seeds of* L. apetalum*. Lepidiumuridine A was evaluated for its estrogen-like effect of* in vitro* and* in vivo*, and we explored its potential mechanisms in this study.

## 2. Materials and Methods

### 2.1. General Experimental Procedure

NMR spectroscopy was performed at room temperature using a Bruker Avance III 500 MHz spectrometer with tetramethylsilane (TMS) as a standard. Optical rotations were measured using an AP-IV laboratory polarimeter, which was made by Rudolph Research Analytical. HRESIMS spectra were determined with a Bruker Maxis HD mass spectrometer. The IR spectrum was measured on a Nicolet iS10 microscope spectrometer, which was made by Thermo Scientific. UV spectra were measured using a Shimadzu UV-2401PC apparatus. P-HPLC was acquired on YMC-Pack ODS-A column (250 × 10 mm and 5 *μ*m, respectively) on a Saipuruisi LC-50 instrument with a UV200 detector. CC was performed on Diaion HP-20 adsorbent (Mitsubishi Chemical Co.), Toyopearl HW-40, MCI gel CHP-20 (Tosoh Co.), Lichroprep RP-18 gel (Merck, Darmstadt), and silica gel (Marine Chemical Industry). TLC was performed on custom silica gel G plates (Qingdao Marine Chemical Industry). The chemical reagents used for isolation were of analytical grade, and the solvents used for* p*-HPLC were of chromatographic grade.

### 2.2. Plant Materials

The seeds of* Lepidium apetalum *Willd. were collected in Xixia county, Nanyang city, Henan province, China, in June 2014. The plant material was identified by Professor Suiqing Chen and Chengming Dong (Henan University of Chinese Medicine), and a voucher specimen (No. 20141101A) was deposited in the university.

### 2.3. Extraction and Isolation

The processed seeds of* L. apetalum* (8.0 kg) were extracted with H_2_O (80L×1.5h×3, 100°C). The aqueous extracts (1.04 kg) was obtained by removing the solvent under reduced pressure; then precipitated at the ethanol concentration of 80%, and the liquid supernatant was concentrated in a vacuum evaporator to yield a gross extract (628 g) that was suspended in H_2_O (1.5 L). The water-soluble substances were resolved on a Diaion HP-20 macroporous resin column and successively eluted with EtOH-H_2_O (0:100, 20:80, 40:60, and 60:40) to obtain 4 fractions (F1-F4). F3 (89.6 g) was suspended in H_2_O and chromatographed on Toyopearl HW-40 CC eluting with MeOH-H_2_O (0:100, 10:90, 20:80, 30:70, and 100:0) to receive fractions (F3.1-3.5). F3.2 was applied to RP-C-18 CC eluted with MeOH-H_2_O (0%→ 100%) to yield F3.2.1-3.2.11. F3.2.3 was purified by semipreparative HPLC with CH3CN: 0.03% CF_3_COOH-H_2_O (10:90, v/v) to afford compound** 1** (200 mg).

Lepidiumuridine A** (1):** colorless crystalline powder; [*α*]_D_^20^ = 35.14 (c 0.35, MeOD); UV (MeOH) *λ*_max_: 206 (2.87), 259 (2.46) nm; IR (iTR) *ν*_max_cm^−1^: 3336, 2925, 1673, 1267, 1033 cm^−1^; HR-ESI-MS: 429.1106 [M+Na]^+^, C_15_H_22_N_2_O_11_; NMR data in Table 1.

### 2.4. Animals

Young female mice, 9-11g, were purchased from the Beijing Vital River Laboratory Animal Technology Co., Ltd. (Beijing, China, certificate No. SYXK2010-004). Forty mice were divided into control group, estradiol valerate group (EV, 0.33 mg/kg, positive control for animal experiments), and two doses of LA (25 and 50 mg/kg). The mice received LA by continuous gavage for 7 d. The animals were euthanized, and blood was collected by heart punctures, and uteri were removed and quickly weighed.

### 2.5. Western Blot

Uterine proteins were extracted using a mammalian protein extraction kit (Beijing Com Win Biotech Co., Ltd.) and quantified with a Bradford protein assay kit (Pierce, Perbio Science Co.). Protein samples separated by SDS-PAGE were transferred to a PVDF membrane. The PVDF membrane was then incubated with a primary antibody (ABclonal, Boston, USA; ER*α* 1:500, A0296; ER*β* 1:500, A2546; GPR30 1:500, A10217) overnight at 4°C and subsequently cultivated with a secondary antibody (1:1000) for 1 h at 25°C. The proteins from the bands were quantified with a chemiluminescence gel imaging apparatus (Azure c500), and the bands were analyzed with Quantity One software.

### 2.6. ELISA

The levels of E2, FSH, and LH (R&D Systems, Minneapolis, MN, USA) in serum were detected by ELISA on the basis of the instructions of manufacturer.

### 2.7. The Effects of LA on MCF-7 Cell Proliferation

MCF-7 cells were cultured in 96-well plates in hormone-free medium with 10% (v/v) charcoal-stripped fetal bovine serum. Cells were treated with 17*β*-E2 (1 *μ*M, positive control drug for cell experiments) and then LA for 24 h; then cell viability was subsequently detected by MTT.

### 2.8. Effects of ICI182780, MPP, THC, and G15 on LA-Promoted MCF-7 Cell Proliferation

Before treatment of 17*β*-E2, ICI182780 (ER-nonspecific antagonist), MPP (Specific ER*α* antagonist), THC (Specific ER*β* antagonist), and G15 (Specific GPR30 antagonist, 1*μ*M, respectively) were added and kept for 0.5h with LA. Other experimental steps are as described above.

### 2.9. In-Cell Western

The cells seeded in 96-well plates were fastened with 4% paraformaldehyde for 20 min at RT and permeabilized by PBS (5 time × 5 min in 0.1% Triton X-100). Cell monolayers were blocked for 1.5h and then cultured with diluted primary antibodies in blocking buffer (1:200) for 2 h at RT. Washing with PBS-T buffer later, the cell layers were stained with IRDyeIgG (1:500) for 1 h and subsequently rinsed and scanned using an Odyssey Infrared Imager. The relative protein expression level was normalized against DRAQ5.

### 2.10. Statistical Analysis

The data were examined by analyzing variance (ANOVA) and SPSS 20.0 software, and the results were expressed as the mean ± SD.

## 3. Results

### 3.1. Compound Identification and Structural Elucidation

**Figure 1:**
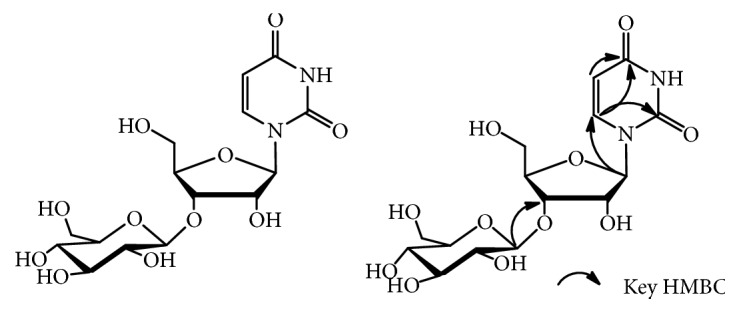
Structure and selected HMBC correlations of compound** 1**.

Compound** 1**, obtained as a colorless crystalline powder, gave the molecular ion peak [M+Na]^+^ at* m/z* 429.1106 in the HRESIMS, which was consistent with the molecular formula C_15_H_22_N_2_O_11_Na (calcd. 429.1121). The IR spectrum presented the absorptions attributed to hydroxyl group (3336 cm^−1^) and carbonyl group (1673 cm^−1^) functionalities. For the ^1^H NMR spectrum of** 1** (Table 1), it showed one* cis*-olefins group protons [8.02 (1H, d,* J* = 8.0 Hz, H-6) and 5.78 (1H, d,* J* = 8.0 Hz, H-5)]. Two anomeric proton signals [*δ *5.87 (1H, d,* J* = 3.0 Hz, H-1′) and* δ *4.41 (1H, d,* J* = 7.5 Hz, H-1′′)] were observed and proved the existence of two *β*-glycosyl. The ^13^C NMR spectrum displayed a total of 15 carbons, combined with the DEPT and HSQC spectra, which were identified as two carbonyl units [*δ*_C_ 166.2 (C-4) and 152.3 (C-2)], two olefins carbons [142.6 (C-6) and 102.3 (C-5)], one ribose carbon signals [91.0 (C-1′), 75.6 (C-2′), 78.7 (C-3′), 84.1 (C-4′), and 61.7 (C-5′)], and one glucopyranosyl unit signals [104.4 (C-1′′), 74.6 (C-2′′), 78.0 (C-3′′), 71.1 (C-4′′), 77.8 (C-5′′), and 62.3 (C-6′′)]. Compared with uridine [16], the NMR spectral data of** 1** only added a glucose unit [17], and the glucose unit was connected to C-3′ of ribose according to glycosidation shift (*δ *78.7 from* δ *75.0). Based on the HMBC correlation of H-1′′ with the C-3′, the assumption was confirmed. In addition, the presence of uridine group and D-glucopyranosyl unit was determined by acid hydrolysis and TLC compared with the standards of uridine and D-glucose. Accordingly,** 1** was characterized as uridine-3′-O-*β*-D-glucopyranoside [18], which is a new natural compound, namely, lepidiumuridine A (Figure 1).

### 3.2. The Effects of Lepidiumuridine A (LA) on Mice

In Table 2, as a positive control, EV increased the uterine coefficient (107.24%) and the levels of FSH, LH, and E2 in serum of mice. The different doses of LA increased the uterine coefficient (41.24% and 47.95%, respectively) and the levels of FSH and E2. See Table 2.

### 3.3. Effect of LA on the Uterine Expression of ER*α*, ER*β*, and GPR30

**Figure 2:**
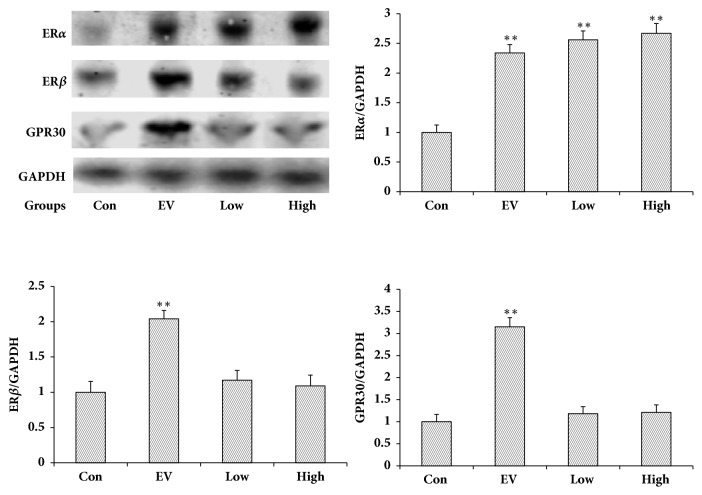
Effect of LA on the uterine expression of ER*α*, ER*β* and GPR30 (n = 3). Low, High: low and high dose of LA. ^*∗∗*^*P*< 0.01 compared to the control group.

Figure 2 shows that although LA promoted the expression of ER*α* in the uterus, it had no effect for ER*β* and GPR30. EV promoted the expression of three proteins in the uterus.

### 3.4. Influences of LA on MCF-7 Cells

**Figure 3:**
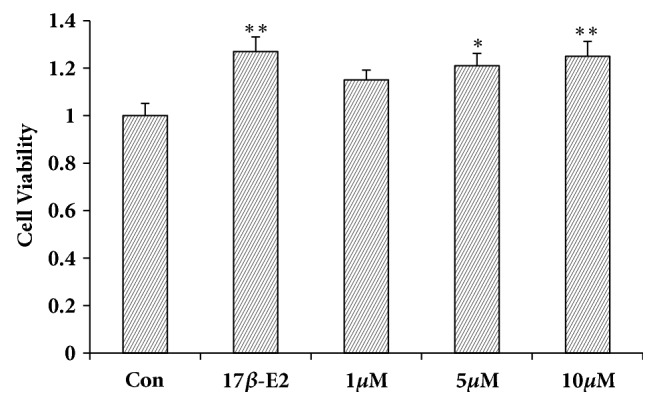
The influence of MCF-7 cell proliferation. Data represent the mean ± SD, n=4; ^*∗*^*P*< 0.05; ^*∗∗*^*P*< 0.01.

Figure 3 shows that LA (different doses) and 17*β*-E2 (as a positive control) promoted MCF-7 cell proliferation.

### 3.5. Effect of MPP on LA-Stimulated MCF-7 Cell Proliferation

**Figure 4:**
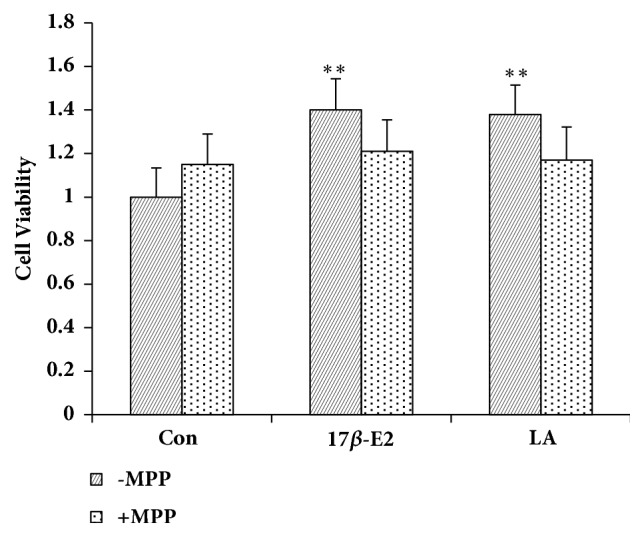
Effect of MPP on MCF-7 cell proliferation. The MPP were appended 0.5h before treatment of 17*β*-E2 and LA. Data represent the mean ± SD, n=3; ^*∗∗*^*P*< 0.01.

As shown in Figure 4, MPP (specific ER*α* antagonist, 1 *μ*M) blocked the effect of LA (10 *μ*M) on MCF-7 cell proliferation.

### 3.6. Effect of MPP on LA Stimulated the Expression of ER*α* in MCF-7 Cells

**Figure 5:**
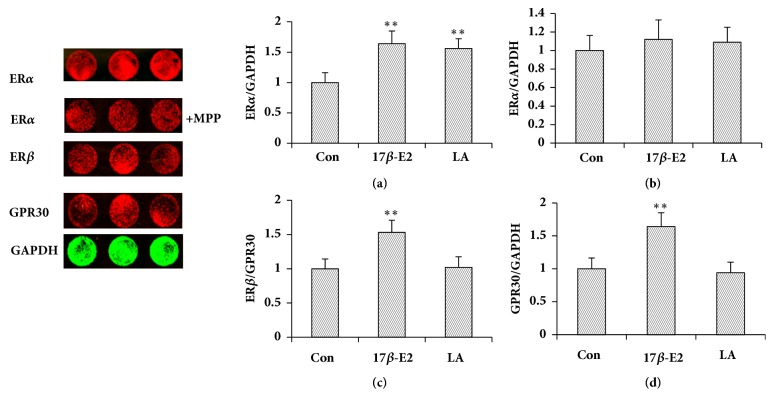
In-cell western tested the expression of ER*α* in MCF-7 cells. Data represent the mean ± SD, n=4; ^*∗*^*P*< 0.05; ^*∗∗*^*P*< 0.01.

The expression of ER*α* in MCF-7 cells induced by LA (10 *μ*M) with the antagonists MPP (1 *μ*M) was displayed in Figure 5. LA can stimulate the expression of ER*α*, but MPP inhibited the LA-stimulated expression of ER*α* in MCF-7 cells.

## 4. Discussion

Phytoestrogens are polyhydric compounds obtained from plants with a structure close to that of mammalian steroid hormone 17*β*-estradiol [19]. Phytoestrogens act in a manner similar to that of estrogen and first bind to estrogen receptors (ERs) [20]. When the ERs bind to ligands, they induce the creation of ER homo- or heterodimers, which in turn stimulate nuclear and extracellular signaling pathways to exert estrogen-like effects [21, 22]. Natural phytoestrogens have lower estrogen-like activity than synthetic estrogen, but they are associated with less risk of breast and endometrial cancers and are safer to use [23]. This is why there has been great interest in the effects and molecular mechanisms of Chinese herbal medicines containing phytoestrogens.

The uterus contains a large number of ERs, and estrogen binds to this receptor, which can induce increased levels of uterine target protein (IPs) that manifests as uterine tissue hyperplasia [24] and is measurable by exogenous sources. In our current research, the uterine coefficient of young female mice was made as an index for evaluating estrogen-like activity. The experimental results displayed that LA significantly increased the uterine coefficient of immature mice, suggesting that LA has estrogen-like effects* in vivo*.

MCF-7 cells are estrogen receptor-positive human breast cancer cells that proliferate in reaction to estrogen or estrogen-like activated material, and they are frequently removed to identify estrogen-like activity [25]. The experimental outcomes showed that LA significantly stimulated MCF-7 cells proliferation, suggesting that LA has estrogen-like effects* in vitro*.

There are two types of estrogen receptors, genomic nuclear ER*α* and ER*β* and the nongenomic GPR30 and potentially additional nongenomic receptors [26]. The receptors mediate the physiological and pathological impacts of estrogen, and western blot and in-cell western were utilized to examine ER*α*, Er*β, *and GPR30 expression in the uterus. These receptors were then used to determine the molecular mechanism of the estrogen-like effect of LA. Western blot revealed that EV (0.33 mg/kg) increased ER*α*, ER*β*, and GPR30 protein expression in the uterus significantly. LA (25 and 50 mg/kg) significantly elevated ER*α* expression in the uterus, indicating that LA's estrogen-like effect occurred* in vivo* through ER*α*. In-cell western showed that 17*β*-E2 significantly boosted ER*α*, Er*β, *and GPR30 expression in MCF-7 cells. LA significantly elevated ER*α* expression in MCF-7 cells, which indicated that the estrogen-like effect of LA acted* in vitro* through ER*α*. In addition, the ER*α* antagonist methyl piperidino-pyrazole (MPP) inhibited LA-stimulated MCF-7 cell proliferation and ER*α* expression in MCF-7 cells.

Lepidiumuridine A, a new natural uridine derivative, was isolated from the seeds of* L. apetalum*. We evaluated the estrogen-like effect of LA* in vitro* and* in vivo* and explored its potential mechanisms. Previous investigations demonstrated that uridine derivatives possess significant biological properties, such as anti-HIV [27], antibacterial [28], antiviral activity [29], and anticancer [30], but no previous reports have described their estrogen-like effect. Prior evaluations have revealed that the phytoestrogens found in traditional Chinese medicine mainly consist of flavonoids, coumarins, lignans, terpenes, steroids, and other compounds [31]. The uridine derivative, lepidiumuridine A, was determined to be a new type of phytoestrogen through our study.

## 5. Conclusion

Lepidiumuridine A, as a new natural uridine derivative, was isolated from the seeds of* L. apetalum*. The compound has estrogen-like effect and the effect was mainly mediated by the estrogen receptor ER*α*.

## Figures and Tables

**Table 1:** NMR data for compound 1 in CD_3_OD.

No.	*δ*_C_	*δ*_H_
2	152.3	
4	166.2	
5	102.5	5.78 (1H, d, 8.0)
6	142.6	8.02 (1H, d, 8.0)
1′	91.0	5.87 (1H, d, 3.0)
2′	75.6	4.29 (1H, m)
3′	78.7	4.29 (1H, m)
4′	84.1	4.19 (1H, m)
5′	61.7	3.88 (1H, dd, 2.0, 12.0) 3.68 (1H, dd, 6.0, 12.0)
1^″^	104.4	4.33 (1H, d, 7.5)
2^″^	74.6	3.35 (1H, m)
3^″^	78.0	3.35 (1H, m)
4^″^	71.7	3.28 (1H, m)
5^″^	78.0	3.33 (1H, m)
6^″^	62.3	3.84 (1H, dd, 2.0, 12.0) 3.78 (1H, dd, 6.0, 12.0)

**Table 2:** Effect of LA on uterine coefficient, FSH, LH, and E2 (*x*±*sd*, *n*=10).

Groups	Dose (mg/kg/d)	Uterine Coefficient (%)	FSH (mIU/mL)	LH (mIU/mL)	E2 (p mol/L)
Con	—	0.08949±0.0123	45.32±5.89	4.55±0.54	29.05±6.11
EV	0.33	0.18546±0.0148^*∗∗*^	56.31±5.12^*∗∗*^	5.72±0.58^*∗∗*^	40.16±5.10^*∗∗*^
LA (Low)	25	0.1264±0.0243^*∗*^	56.47±5.76^*∗∗*^	3.89±0.51	35.67±5.17^*∗*^
LA (High)	50	0.1324±0.0364^*∗*^	59.19±6.12^*∗∗*^	4.11±0.35	37.13±4.96^*∗*^

^*∗*^ *P* < 0.05; ^*∗∗*^*P* < 0.01 compared to the control group.

## Data Availability

The data used to support the findings of this study are available from the corresponding author upon request.

## Supplementary Materials

Supplementary Materials The file includes the spectra of 1D and 2D NMR, IR, UV, and MS, and the spectra were used to clarify and determine the structure of lepidiumuridine A.
