# Supplementary material for: Lepidiumuridine A: A New Natural Uridine Derivative as a Phytoestrogen Isolated from the Seeds of Lepidium apetalum Willd
Source: Evid Based Complement Alternat Med. 2018 Sep 4;2018:2813465. doi: 10.1155/2018/2813465 (PMC6142764; doi:10.1155/2018/2813465)
Supplement: Supplementary Materials — The file includes the spectra of 1D and 2D NMR, IR, UV, and MS, and the spectra were used to clarify and determine the structure of lepidiumuridine A. [file 2813465.f1.docx]

Lepidiumuridine A, a new natural uridine derivative as phytoestrogen isolated from the seeds of *Lepidium Apetalum* Willd.

Mengnan Zeng,^1,2,†^ Meng Li,^1,2,†^ Zhi-guang Zhang,^1,2^ Beibei Zhang,^1,2^ Jingke Zhang,^1,2^ Xiaoke Zheng,^1, 2^ Weisheng Feng^1,2^*

*^1^ Henan University of Chinese Medicine, Zhengzhou 450046, China;*

*^2^ Collaborative Innovation Center for Respiratory Disease Diagnosis And Treatment & Chinese Medicine Development of Henan Province, Zhengzhou 450046, China.*

**Abstract:**

Phytoestrogens are polyhydric compounds derived from plants and have a similar structure to 17*β*-estradiol. The present study was to examine the estrogenic effects of lepidiumuridine A (LA), a new natural uridine derivative, which was isolated from the seeds of *L. apetalum*. The structure was elucidated and determined via analysis of extensive spectroscopic data interpretation. The activity of the LA was investigated by the uterine weight gain of mice and a proliferation assay of breast cancer cell lines (MCF-7 cell). Western blot, In-cell western and antagonist assays with methylpiperidino-pyrazole (MPP) were used to explore the mechanism of it. Results showed that LA signiﬁcantly increased the uterus coefﬁcient and the expression of ER*α* in uterus and MCF-7 cells. MPP could inhibit LA-stimulated the MCF-7 cell proliferation and the expression of ER*α* in MCF-7 cells. Taken together, LA had an estrogen-like effect which was mainly mediated by the estrogen receptors ER*α.*

**Keywords:** The seeds of *Lepidium apetalum* Willd.; lepidium[alkaloid](javascript:void(0);) A; uridine derivative; estrogen-like effect; phytoestrogen.


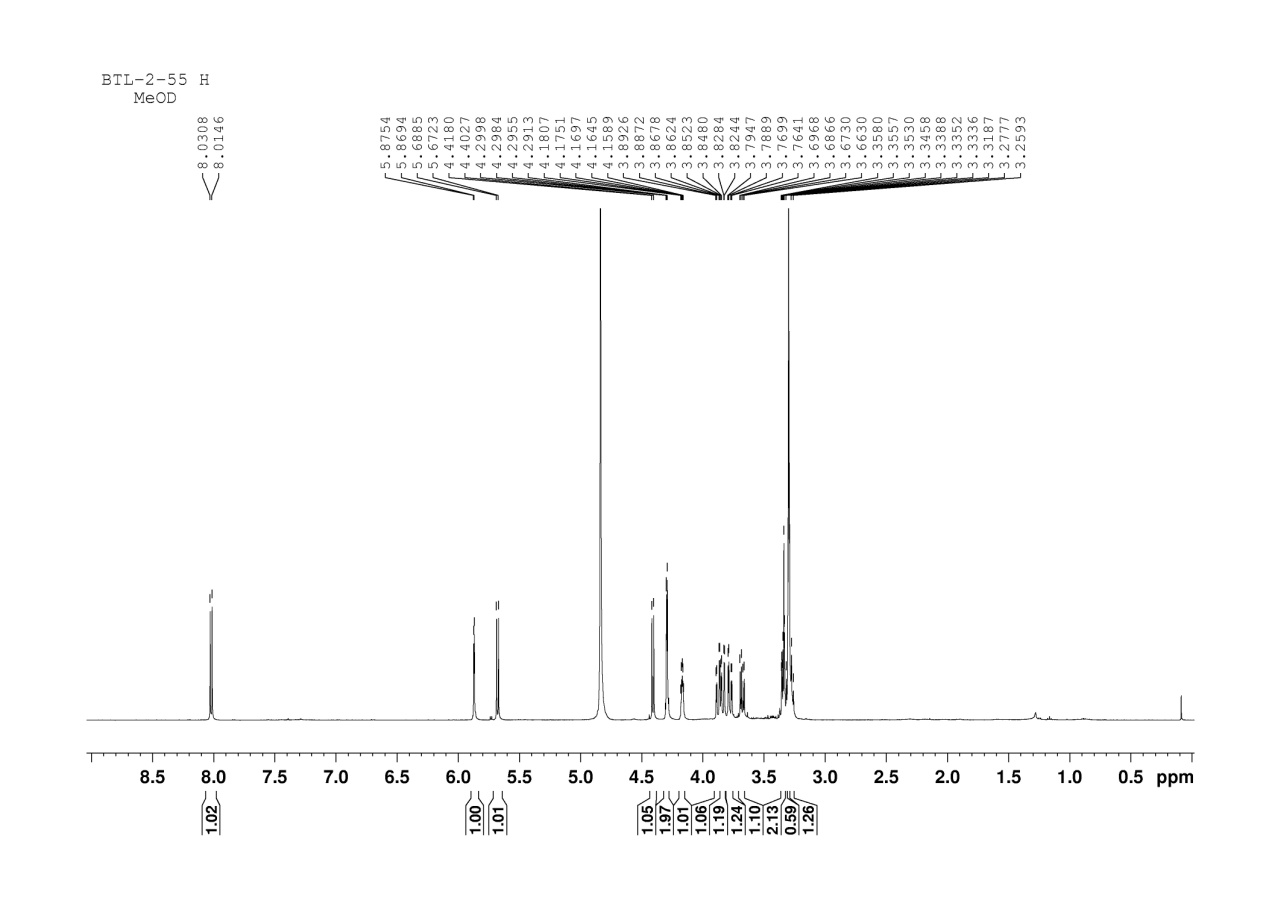


Fig.1S The ^1^H-NMR spectrum of compound **1**（in CD_3_OD）


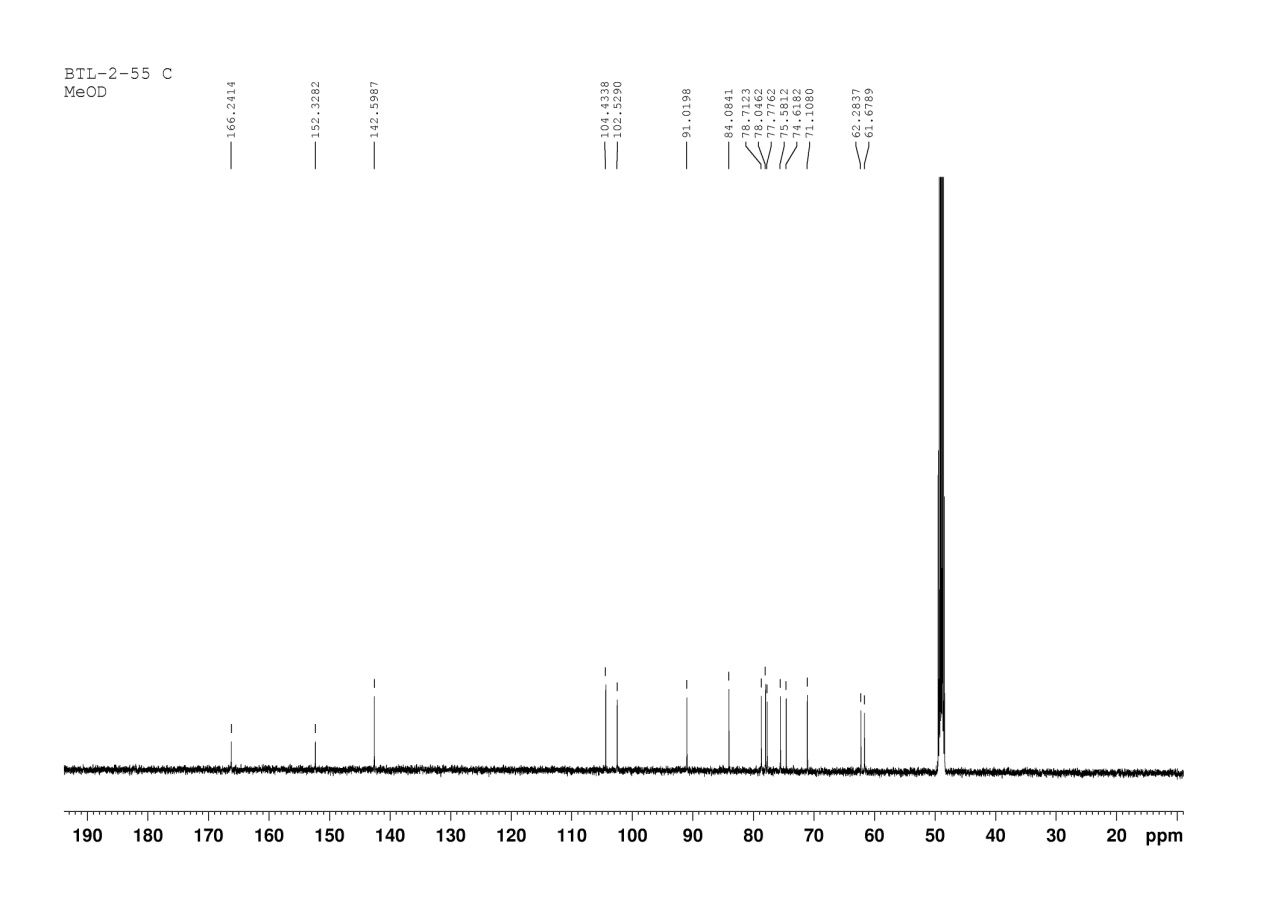


Fig.2S The ^13^C-NMR spectrum of compound **1** （in CD_3_OD）


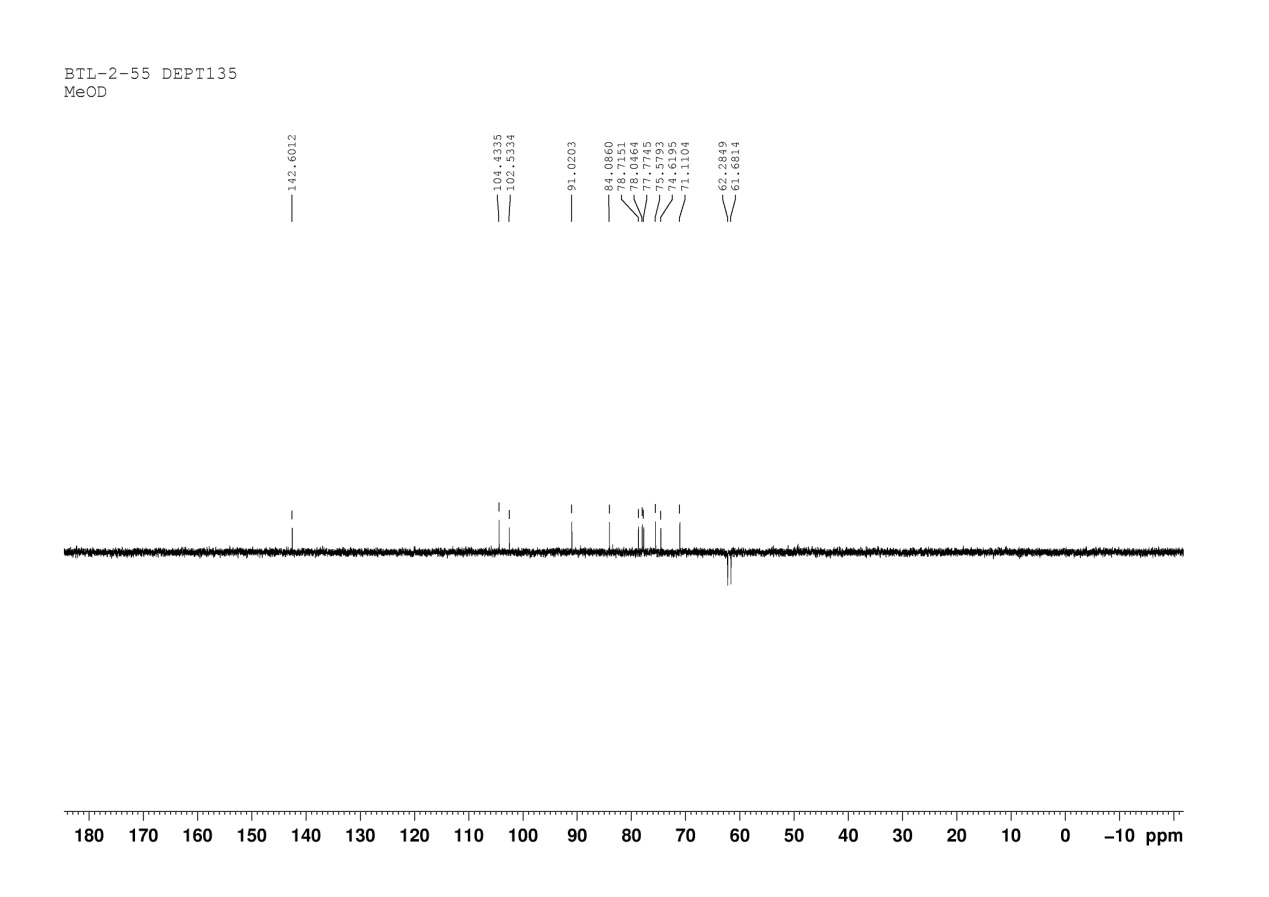


Fig.3S The DEPT 135 spectrum of compound **1**


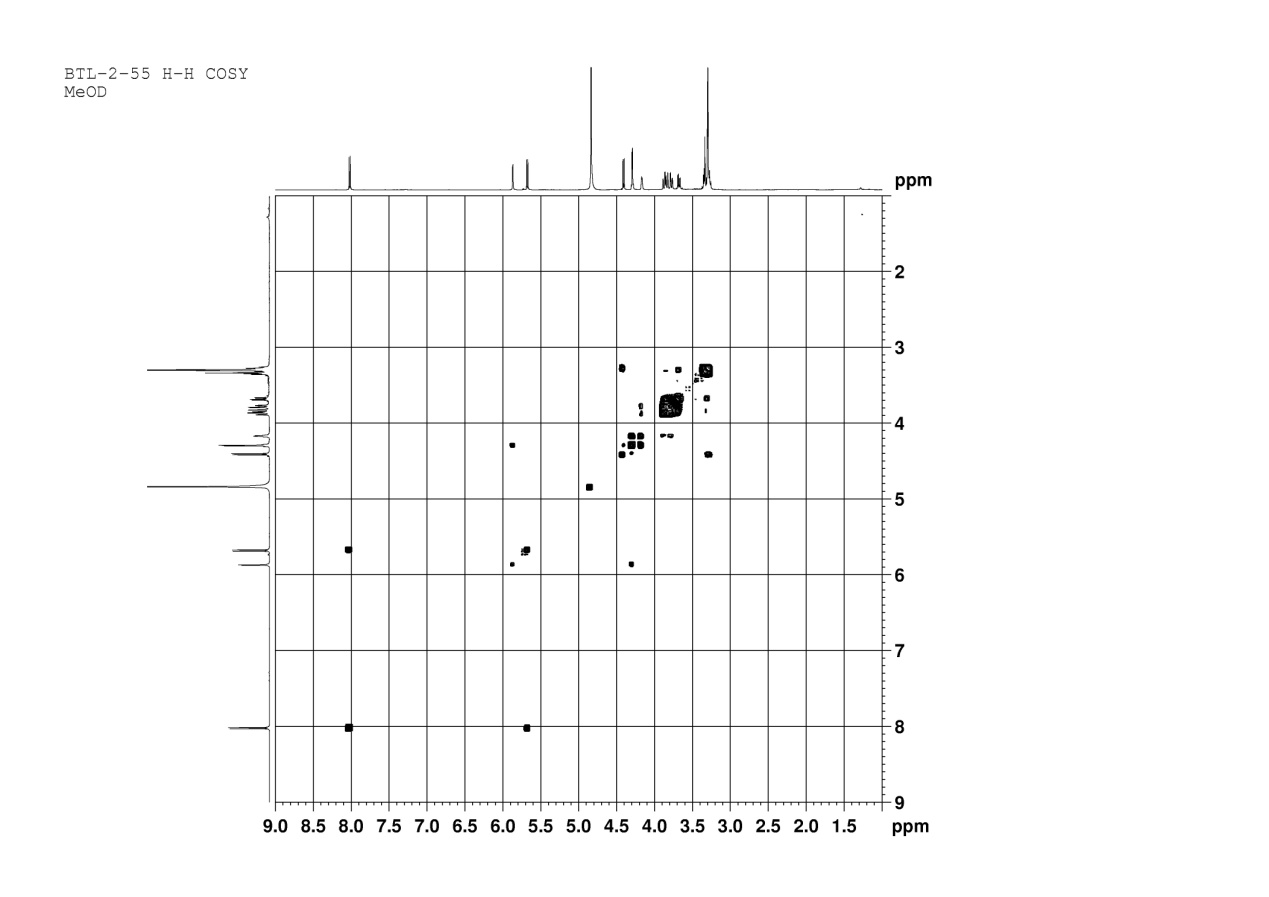


Fig.4S The ^1^H-^1^H COSY spectrum of compound **1**


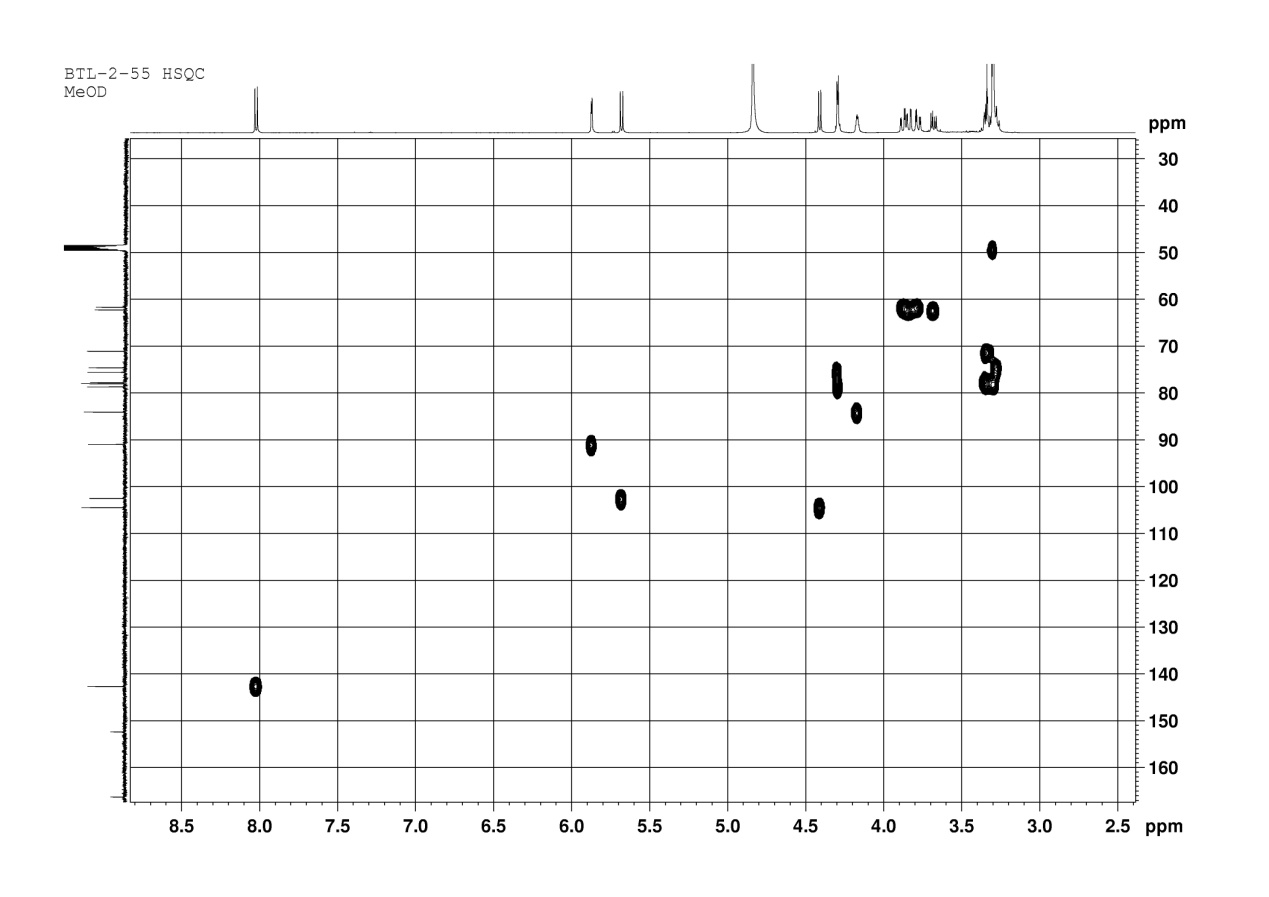


Fig.5S The HSQC spectrum of compound **1**


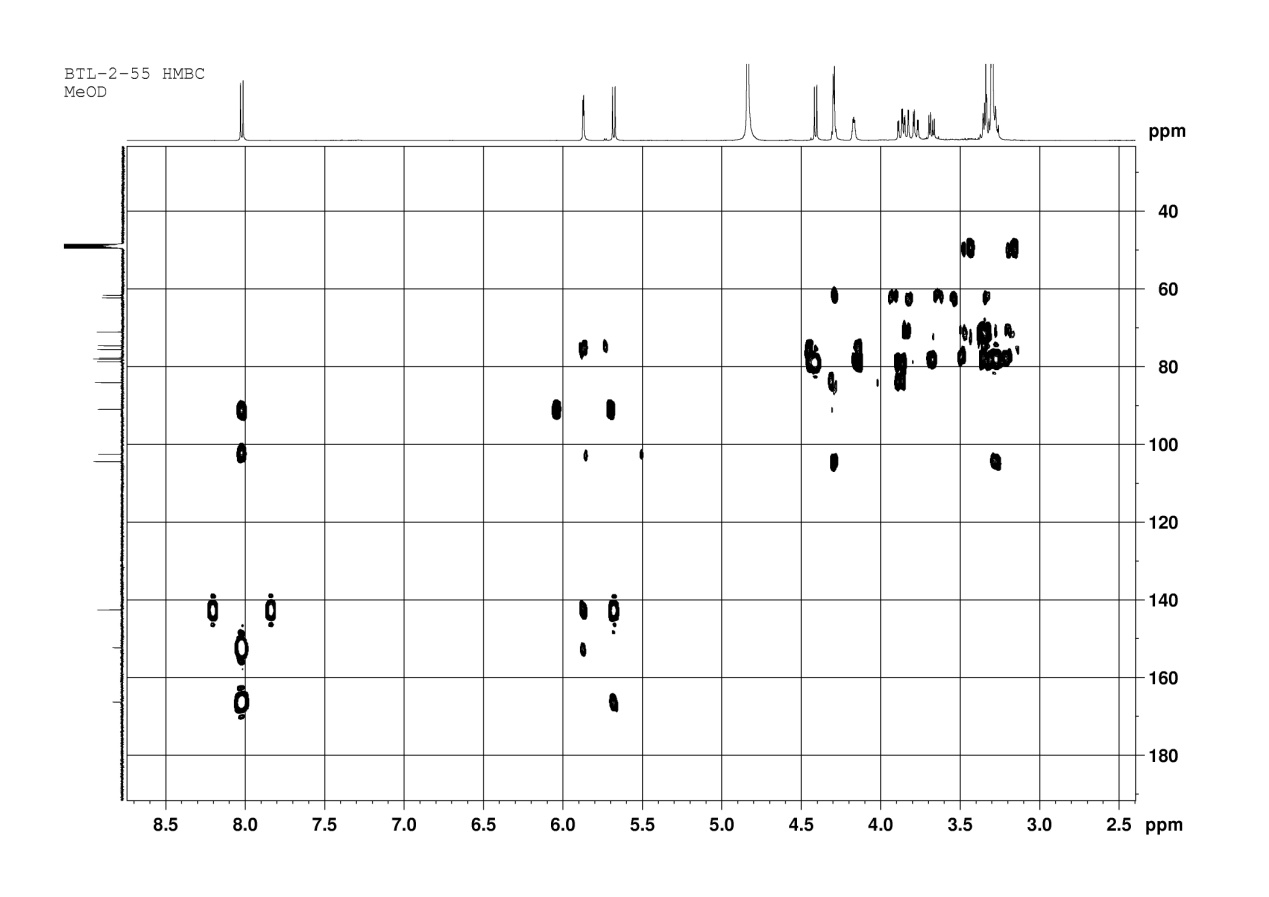


Fig.6S The HMBC spectrum of compound **1**


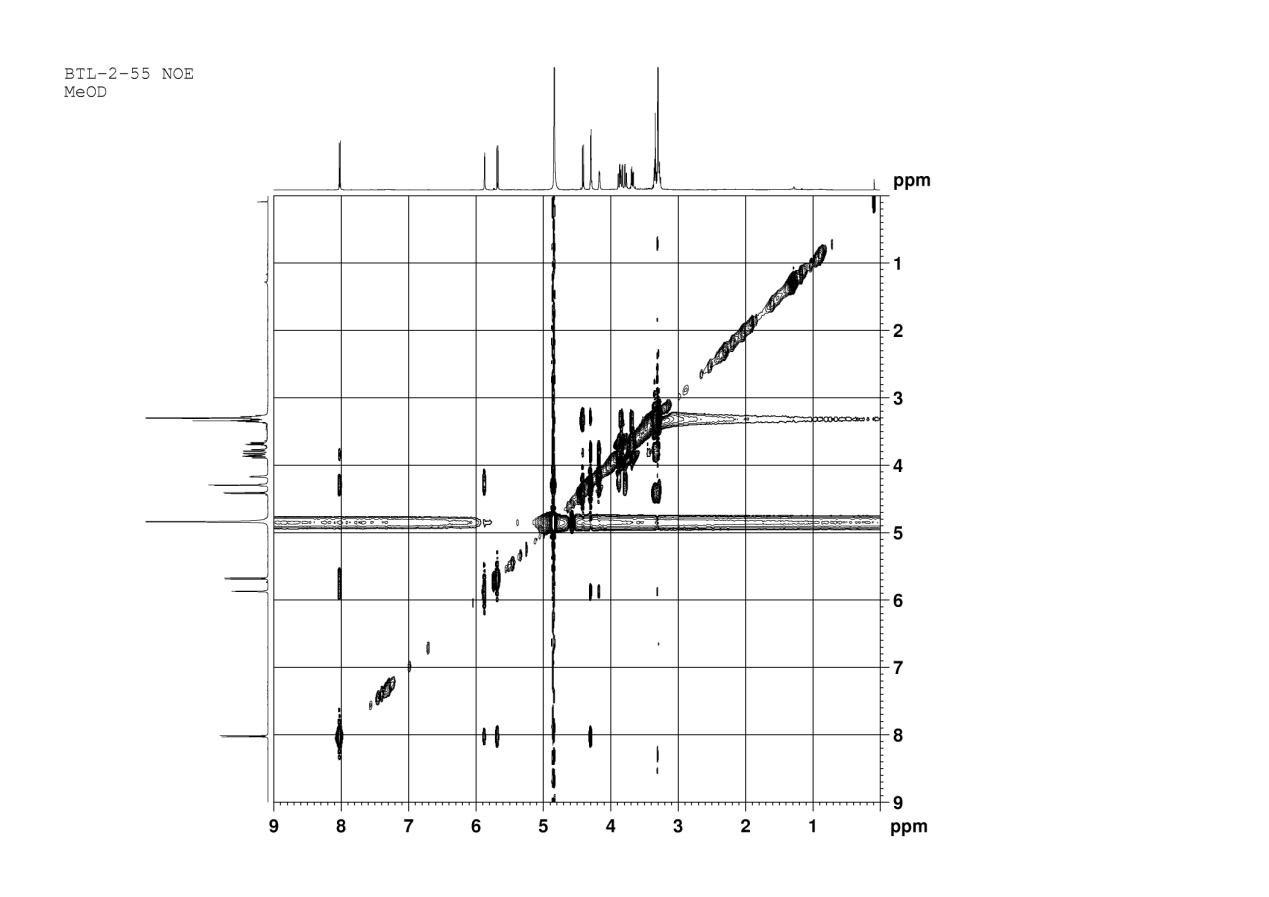


Fig. 2-7 化合物BTL-2-55的NOESY谱


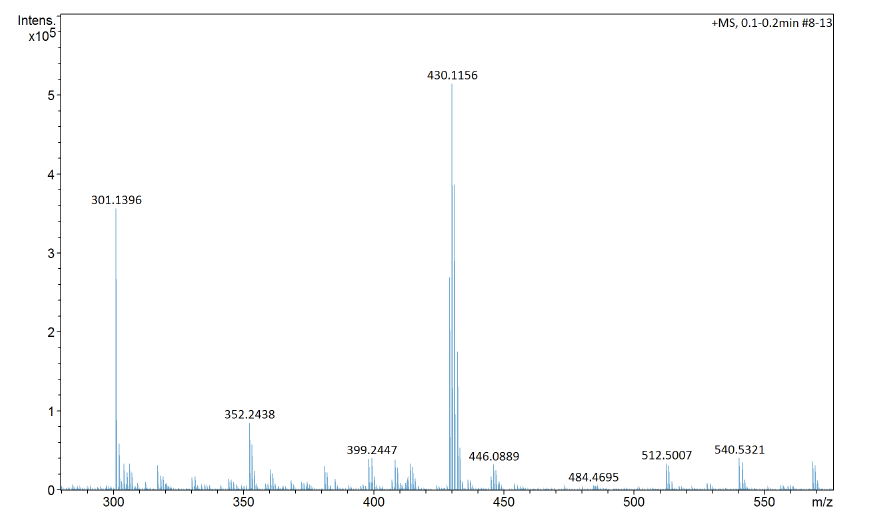


Fig.8S The HR-TOF-MS spectrum of compound **1**


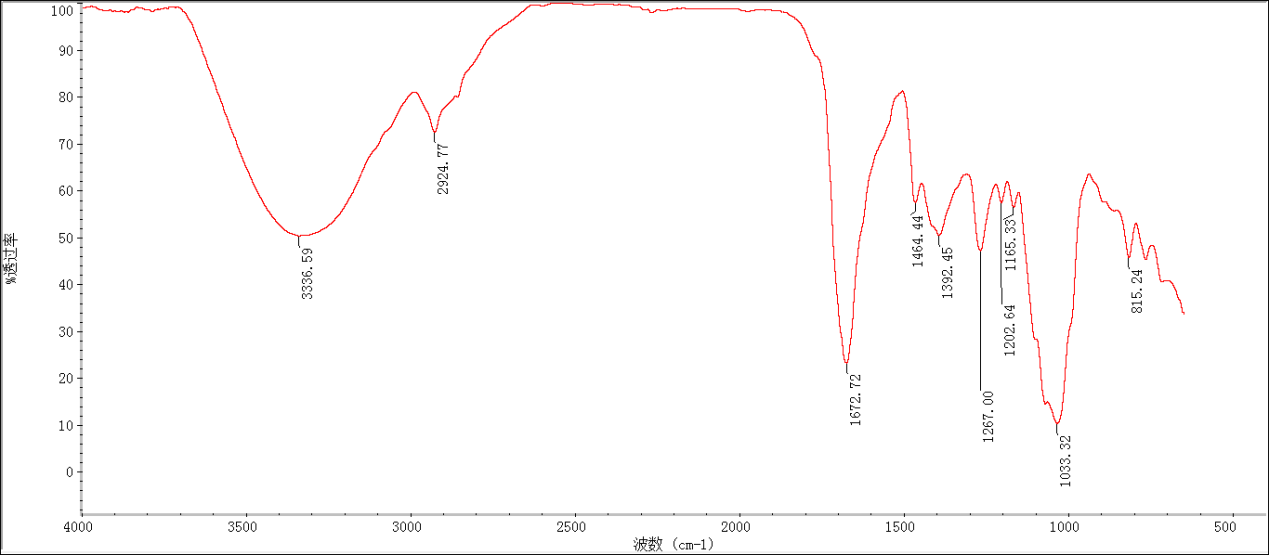


Fig.9S The IR spectrum of compound **1**


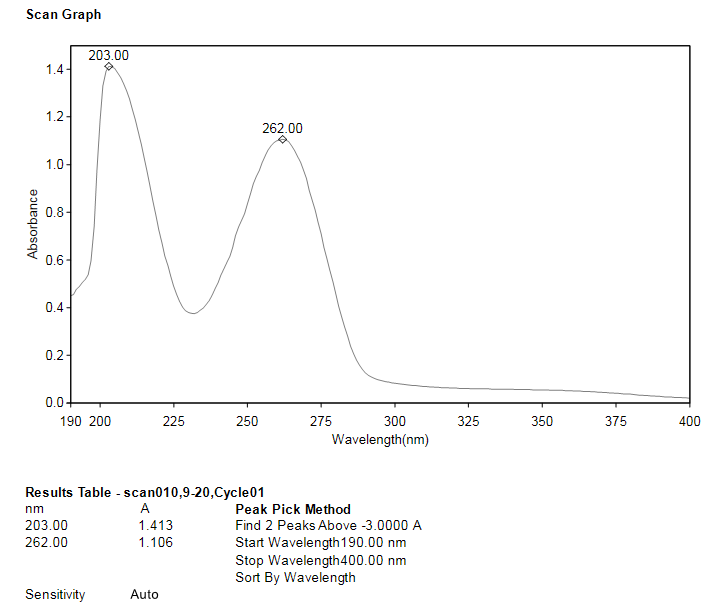


Fig.10S The UV spectrum of compound **1**
